# High Performance Both in Low-Speed Tracking and Large-Angle Swing Scanning Based on Adaptive Nonsingular Fast Terminal Sliding Mode Control for a Three-Axis Universal Inertially Stabilized Platform

**DOI:** 10.3390/s20205785

**Published:** 2020-10-13

**Authors:** Yuanchao Wang, Yongming Yang, Haipeng Kuang, Dongming Yuan, Chunfeng Yu, Juan Chen, Nan Hua, Han Hou

**Affiliations:** 1 Changchun Institute of Optics, Fine Mechanics and Physics, Chinese Academy of Sciences, Changchun 130033, China; wangyuanchao@ciomp.ac.cn (Y.W.); kuanghaipeng@ciomp.ac.cn (H.K.); yuandongming@ciomp.ac.cn (D.Y.); yuchunfeng@ciomp.ac.cn (C.Y.); chenjuan@mail.ccut.edu.cn (J.C.); huanan@ciomp.ac.cn (N.H.); houhan@ciomp.ac.cn (H.H.); 2 Key Laboratory of Airborne Optical Imaging and Measurement, Chinese Academy of Sciences, Changchun 130033, China; 3 University of Chinese Academy of Sciences, No. 19, Yuquan Rd., Beijing 100049, China; 4 School of Electrical and Electronic Engineering, Changchun University of Technology, Changchun 130012, China

**Keywords:** inertially stabilized platform, adaptive control, nonsingular fast terminal sliding mode control, Lyapunov stability theory, uncertain disturbance, input saturation, novel reaching law, low-speed tracking, large-angle swing scanning

## Abstract

In order to improve the performance in the practical engineering applications including so called low-speed video tracking and large-angle swing scanning imaging at the same time for a three-axis universal inertially stabilized platform (UISP), we propose an adaptive nonsingular fast terminal sliding mode control (ANFTSMC) strategy subjected to the uncertain disturbances and input saturation constraints. First of all, a second-order dynamic model is established with uncertain disturbances and input saturation constraints. Secondly, a nonsingular fast terminal sliding mode controller (NTSMC) is constructed to ensure the system error converges to zero fast in a finite time; meanwhile, a novel reaching law based on a modified normal distribution function is designed to adjust the control gain. Thirdly, an adaptive control law is designed to online estimate the parameters of the lumped uncertain disturbances. Additionally, the stability of the control system is proved by Lyapunov theory. Finally, extensive comparative simulations and experiments are carried out, the results comprehensively show the effectiveness and superiority of the proposed control method, which can accelerate convergence, weaken the chattering, and has the better control accuracy and robust performance both in the low-speed tracking and large-angle swing scanning applications. Moreover, the exact dynamic model and the prior knowledge of the upper bounds of the disturbances are not required during the procedure of the controller design, which make it have more extensive application value in practical engineering.

## 1. Introduction

Initially stabilized platform is widely used in the field of modern aerial remote sensing [1,2,3]. Its main function is to isolate payloads from external disturbance and ensure the relative stability of the line of sight (LOS) in inertial space, so as to implement diverse missions such as battle reconnaissance, aerial photography, environmental disaster monitoring, etc. So far, most typical optical stabilized platforms have integrated multiple payloads to satisfy various surveillance and reconnaissance purposes [2,3]. However, the total number of payloads is limited by the structural space; on the other hand, the stacking of multiple payloads is redundant for different customers. In order to broaden the range of applications, a three-axis universal inertially stabilized platform studied in this work is designed as an azimuth axis cantilever structure, thanks to the rotation ranges of azimuth, roll and pitch angles are ±180°, ±80°, and ±45°, respectively, and different payloads can be conveniently installed and replaced by the standard interfaces, the UISP integrates the common characteristics of payloads including low-speed video tracking and large-angle swing scanning imaging, which makes it have advantages of lower cost, higher task efficiency, and wider customer groups.

Due to the influence of external nonlinear factors such as the attitude change of the aviation platform and the dynamic motion of target, the image quality is easily degraded. At the same time, different payloads have different masses and centroids which are difficult to accurately measured, so it is a great challenge to obtain an exact dynamic model and design an effective control algorithm that takes into account both the two applications under the different payloads. The traditional linear proportional-integral-derivative (PID) controller [4,5,6] has been widely used in the motor drives due to its simplicity in implementation. However, with the increasing requirements of information task, higher positioning and tracking performances are needed to adapt to some special applications. At this time, PID controller often hit a bottleneck. Therefore, many advanced control methods such as adaptive control (AC) [7,8,9,10], sliding mode control (SMC) [11,12,13,14,15], neural network control (NNC) [10,16,17], fuzzy control (FC) [6,18,19], active disturbance rejection control (ADRC) [20,21], Disturbance Observer-Based Control [22,23], and Extended State Observer (ESO) [7,24,25] have been studied to deal with nonlinear and uncertain problems. Among them, SMC has been widely praised in nonlinear control systems because of its good dynamic performance, insensitive to nonlinear disturbances and robustness to parameter perturbation. After depth analysis, we know that the traditional linear sliding mode control can only achieve asymptotic control error, and it needs high gain to achieve fast convergence, which inevitably leads to the high frequency dynamic behavior and life loss of the actuator, over time, it may lead to the instability. In order to overcome this weakness, nonlinear functions can be constructed appropriately in the sliding surface and controller, for example, the terminal sliding mode control (TSMC) of nonlinear sliding surface is developed in [11]. However, due to the existence of negative fractional power term, this kind of method has singular problems and leads to infinite control input. Subsequently, nonsingular terminal sliding mode control (NTSMC) is proposed in [12], which can solve the singular problem well by constructing a control law with constrained fractional power; however, the prior knowledge of the upper bound of the system uncertainty is required. In the practical applications, it is necessary to conservatively estimate a sufficiently large constant for the uncertain upper bound, which will increase the control quantity and cause unnecessary chattering accordingly. Up to now, there are generally two methods to solve the chattering, one is to employ a continuous function such as smooth hyperbolic tangent function to replace symbolic function [26], the other one is to adjust switching gain by designing a new reaching law [3,27,28]; it is worth mentioning that the latter can also shorten the convergence time when the system state is far away from the equilibrium state.

As we all know that the adaptive control (AC) can estimate the uncertainty online by constructing an appropriate adaptive law without prior knowledge of the upper bound of uncertainty [7,8,9,10,15], which can improve the robustness of the control system. Because of this, the hybrid control of AC and SMC does not need high gain to obtain fast convergence performance, which can effectively solve the chattering problem and has great potential to deal with nonlinear disturbance and uncertainty. Based on this point, the adaptive nonsingular fast terminal sliding mode control (ANFTSMC) integrates all advantages of the AC and NTSMC methods, and becomes a new research hotspot [8,9,15,16,18,21,23,26,29,30,31,32,33,34,35,36,37,38]. Although these algorithms have achieved good results, there are still some problems to further studied: (i) Most methods [9,15,16,18,21,26,29,31,34] have been demonstrated only in the simulations but not applied in the practical systems under input saturation constraint; (ii) Some methods [8,23,30] depend on the dynamic model, resulting in poor applicability under disturbances; (iii) The researchers rarely have clarified the convergence time after introducing adaptive control; (iv) These works are difficult to achieve high performance both in the low-speed tracking and large-angle swing scanning applications. It is worth noting that a good solution to the second problem in [7], the authors designed an adaptive sliding mode control based on the ESO for an electro-mechanical servo system with unknown friction and input saturation constraint.

Motivated by above methods, this paper takes a three-axis UISP which can be conveniently installed and replaced different payloads as the research object. Aiming at the practical control problems in the low-speed video tracking and large angle swing scanning imaging applications, a composite control method based on NFTSMC and AC is proposed to achieve the UISP position tracking of the reference signal. The control method overcomes the chattering and singularity problems of the traditional sliding mode, ensures the fast convergence and robustness, and improves the control accuracy of the servo system. At the same time, considering the input saturation constraint of the actual system, the effectiveness and robustness of the proposed control method are verified by a large number of simulations and experiments.

The main contributions of this paper are given as follows:

(i) The proposed method does not rely on an exact dynamic model, where the friction, imbalance and unmodelled dynamics are regarded as parts of the lumped uncertain disturbance. Then the lumped disturbance is designed as a function of angular position and speed. The adaptive control law is designed to online estimate the parameters of the lumped uncertain disturbance. What is more potential from the practical application point of view is that the prior knowledge in the upper bounds of the uncertainties is not required during the procedure of the controller design.
(ii) Based on the nonsingular fast terminal sliding mode control theory, a novel reaching law is designed in this paper, to overcome the chattering and singularity problems in the traditional sliding mode control; meanwhile, it not only can accelerate convergence in a finite time when the system state is far away from the equilibrium, especially in the large-angle swing scanning application, but also further weaken the chattering during the system state maintain on the sliding surface.
(iii) A smooth hyperbolic tangent function is designed to approximately replace the non-smooth input saturation, the approximate error is regarded as part of the total disturbance, makes it more convenient in practical implementation.
(iv) The proposed method can improve the performance of the low-speed video tracking and large-angle stepping swing scanning applications at the same time, it has more extensive application value in practical engineering.

The remainder of this paper is organized in the follow manner. First, in Section 2, the three-axis UISP studied in this paper is described. Second, in Section 3, the problem statement and preliminaries about dynamic are introduced. Third, Section 4 is devoted to design the composite controller based nonsingular fast terminal sliding mode control and adaptive control theory, in addition, stability analysis based on Lyapunov theory is also given in this section. Then, in Section 5, extensive simulations and practical experiments are given to analyze the effectiveness of the proposed composite controller. Finally, conclusions are given in Section 6.

## 2. System Description

**Figure 1:**
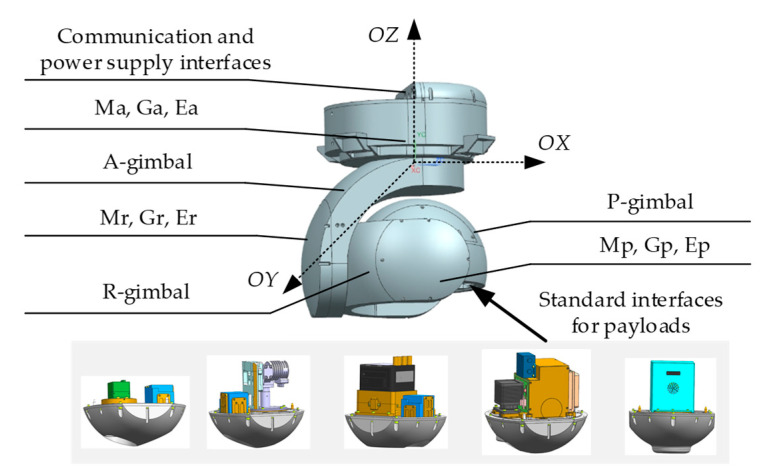
Schematic diagram of the three-axis universal inertially stabilized platform.

As shown in the Figure 1, the three-axis universal inertially stabilized platform studied in this paper consists of three gimbals, the A-gimbal, R-gimbal, and P-gimbal. Among them, the P-gimbal is assembled on the R-gimbal, and can rotate around the OY axis. Similarly, the R-gimbal is assembled on the A-gimbal, and can rotate around the OX axis. The A-gimbal is assembled on the base, and can rotate around the OZ axis. A three-axis gyroscope and position and orientation system (POS) are mounted on the inner P-gimbal of the UISP. And the standard interfaces are assembled on the pitch shafting in order to realize the installation and replacement of different payloads. In order to achieve the composite applications of the UISP with various payloads, including video tracking, swing scanning and attitude compensation, the gyro and encoder are indispensable. G_a_, G_r_, and G_p_ stand for the gyroscopes, which are used to measure the inertial angular rate of the A-gimbal, R-gimbal, and P-gimbal, respectively. E_a_, E_r_, and E_p_ stand for the encoders are used measure the relative angular between the gimbals, respectively. M_a_, M_r_, and M_p_ stand for gimbal servo motors that drive the A-gimbal, R-gimbal, and P-gimbal, respectively. When the aviation platform rotates or jitters, the control system of the UISP compensates the attitude information measured by the POS to keep the LOS of imaging sensors relative stable in inertial space.

## 3. Problem Statement and Preliminaries

In the three-axis UISP, each gimbal is driven by its own DC torque motor, the motor rotator is directly mounted on the load shaft, which indicates that the rotator and the load shaft are fixed as a rigid body and improves the coupling stiffness of the system. Meanwhile, the encoder, gyro and motor are designed to be coaxial and calibrated during installation. So, the cross coupling effect of the three axis is small, and the motion of each axis can be decoupled and considered separately.

To facilitate the control design, ignoring the cross coupling between shafting, for each axis of the UISP, the second-order dynamic model with mass imbalance, gimbal friction, aircraft attitude turbulence, model uncertainty, and input saturation can be described as below.

(1) $$J\ddot{\theta }\;=K_{t}\Phi (u)-K_{e}\dot{\theta }-T_{f}-T_{g}-T_{d}$$

where θ, $\dot{\theta }$, and $\ddot{\theta }$ are the motor angular position, velocity, and acceleration, respectively. All of them are bounded, θ and $\dot{\theta }$ can be measured in real-time; J and $K_{e}$ are the equivalent moment of inertia and damping coefficients on the motor shaft side; $K_{t}$ stands for the constant coefficient of motor torque; $T_{f}$ denotes the friction torque; $T_{g}$ is the imbalance torque; $T_{g}$ represents the external disturbances; and u is the control input to the actuator. Φ(u) denotes the output of the saturation. Without loss of generality, assuming Φ(u) has symmetry and can be given by

(2) $$\Phi (u)=\left\{ \begin{array}{ll} \varphi_{\max}\mathrm{sign}(u) & \mathrm{if}\;|u|\;\geq \;u_{\mathrm{sat}}\\ u & \mathrm{if}\;|u|\;<\;u_{\mathrm{sat}} \end{array}\right.$$

where $\varphi_{\max}$ is the maximum output of the actuator, $u_{\mathrm{sat}}$ is the input value of critical saturation, and sign(·) represents the sign function.

The above non-smooth saturation dynamic cannot be directly used in the control design and synthesis, in particular for adaptive control. Hence, the saturation function Φ(u) can be approximated by the following smooth hyperbolic tangent function g(u) as [7]

(3) $$g(u)=\varphi_{\max}\times \tan h(\frac{u}{\varphi_{\max}})$$

**Figure 2:**
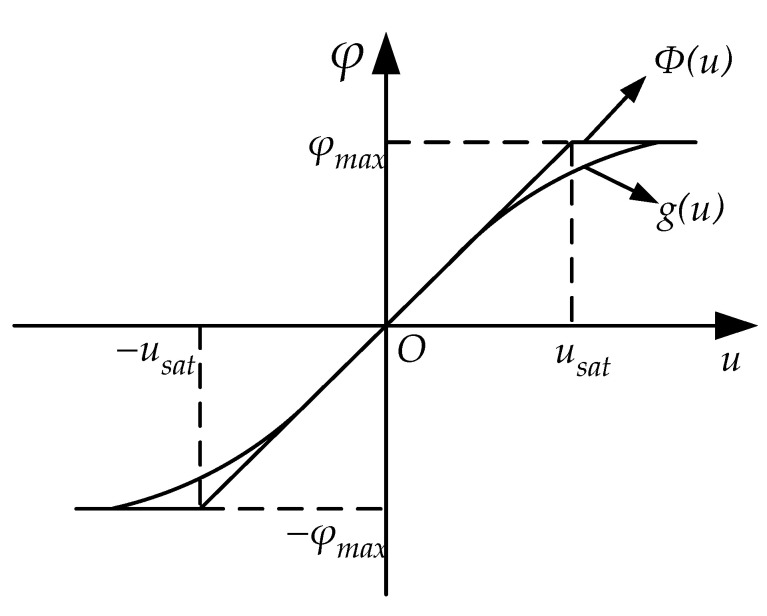
Non-smooth saturation and smooth hyperbolic tangent saturation.

As shown in Figure 2, the non-smooth saturation can be approximated by a smooth hyperbolic tangent function tanh (·), then Equation (2) can be expressed as

(4) Φ(u)=g(u)+δ(u)

where δ(u) is bounded satisfying

(5) $$|\delta (u)|\leq D_{1}$$

where $D_{1}$ is a bounded positive constant.

By using the mean-value theorem as [7], for any $u_{0}$ there exists a constant 0<ξ<1, such that

(6) $$g(u)=g(u_{0})+g_{u_{\xi }}(u-u_{0})$$

where $g_{u_{\xi }}=\frac{\partial g(u)}{\partial u}{|}_{u=u_{\xi }}$ is a bounded function of $u_{\xi }$ given by $u_{\xi }=\xi u+(1-\xi )u_{0}$. By choosing $u_{0}=0$, then $g(u_{0})=0$, and g(u) can be expressed in a linear form as

(7) $$g(u)=g_{u_{\xi }}u$$

It comes naturally that Equation (4) can be mathematically formulated as a linear-like system of u with the time-varying gain $g_{u_{\xi }}$ and a bounded disturbance ε(u) given by

(8) $$\Phi (u)=g_{u_{\xi }}u+\varepsilon (u)$$

Hence, the dynamic model Equation (1) can be rewritten as

(9) $$J\ddot{\theta }=K_{T}u-K_{e}\dot{\theta }-T_{f}-T_{g}-T_{g}+\varepsilon_{1}$$

where, $K_{T}=K_{t}g_{u_{\xi }}$ and $\varepsilon_{1}=K_{T}\varepsilon (u)$.

In practice the dynamic parameters can be expressed in term of a known nominal part and unknown or uncertain part, as follows

(10) $$\begin{array}{l} J=J^{0}+\Delta J\\ K_{T}=K_{T}^{0}+\Delta K_{T}\\ K_{e}=K_{e}^{0}+\Delta K_{e} \end{array}$$

where $J^{0}$, $K_{T}^{0}$ and $K_{e}^{0}$ are the nominal parts, ΔJ, $\Delta K_{T}$ and $\Delta K_{e}$ are the uncertain parts induced by unmodelled dynamics and perturbation of the different payloads’ parameters. So, the dynamic model Equation (9) can be rewritten as

(11) $$\ddot{\theta }=A_{0}\dot{\theta }+B_{0}u+\delta_{1}+\delta_{2}+\delta_{3}$$

where, $A_{0}=-\frac{K_{e}^{0}}{J^{0}}$ is the state matrix coefficient, $B_{0}=\frac{K_{T}^{0}}{J^{0}}$ is input gain, $\delta_{1}=\frac{\Delta K_{T}u-\Delta J\ddot{\theta }-\Delta K_{e}\dot{\theta }-T_{f}-T_{g}}{J^{0}}$ is the model uncertainty disturbance, $\delta_{2}=-\frac{T_{d}}{J^{0}}$ is the external disturbance and $\delta_{3}=\frac{\varepsilon_{1}}{J^{0}}$ is the saturation approximation error.

Following, we choose the lumped unknown disturbance δ stands for the sum of model uncertain disturbance $\delta_{1}$, external disturbance $\delta_{2}$, and the saturation approximation error $\delta_{3}$.

Hence, the dynamic model Equation (11) can be rewritten as follows

(12) $$\ddot{\theta }=A_{0}\dot{\theta }+B_{0}u+\delta$$

The objective of our research is to design an appropriate sliding mode controller combining the adaptive theory for the three-axis UISP, so that the output angular position θ can track the reference $\theta_{d}$ in the desired finite time without any singularity and serious chattering, and the robustness of the system is guaranteed from the initial state ${\left[\theta (0),\dot{\theta }(0)\right]}^{T}$. In order to make the remaining work more rigorous, it is necessary to make the following assumptions.

**Assumption 1.**  *The reference angular position*$\theta_{d}$* is twice continuously differentiable in terms of time*t.

Then we can define the angular position tracking error $e_{1}=\theta -\theta_{d}$ and its derivative $e_{2}=\dot{\theta }-{\dot{\theta }}_{d}$. The dynamic model Equation (12) can be represented in the error-state space form as follows

(13) $$\left\{ \begin{array}{l} {\dot{e}}_{1}=e_{2}\\ {\dot{e}}_{2}=-{\ddot{\theta }}_{d}+A_{0}\dot{\theta }+B_{0}u+\delta \end{array}\right.$$

**Assumption 2.**  *The input gain* $B_{0}$ *is non-zero constant.*

**Assumption 3.**  *The lumped uncertainty disturbance* δ *is unknown and upper bounded by a positive constant* D *as follows*

(14) |δ| ≤D

If we suppose the upper bound of the lumped uncertainty disturbance is a function containing only angular position θ and velocity $\dot{\theta }$ measurements which have been successfully applied to manipulators [26,29], in this paper, taking $e_{1}$ and $e_{2}$ replace θ and $\dot{\theta }$, respectively, thus D can be compressed as the approximate result of second-order Taylor expansion of the system state error.

(15) $$D=d_{0}+d_{1}||e_{1}||+d_{2}||e_{2}|{|}^{2}$$

where $d_{0}$, $d_{1}$, and $d_{2}$ are all positive constants, and ||·|| stands for Euclidean Norm.

**Assumption 4.**  *The angular velocity tracking error is assumed to be zero at*t=0*, i.e.,*$e_{2}(0)=0$.

## 4. Controller Design

In this section, an ANFTSMC method is proposed to accomplish the control of the UISP system with unknown uncertainty disturbance and input saturation constraints. Firstly, NFTSMC method is designed by three steps. Secondly, AC method is introduced to online estimate the parameters of the lumped uncertain disturbances. Then, in order to accelerate convergence and weaken the chattering, a novel reaching law is designed based on a normal distribution function is designed to adjust the switching gain. Finally, stability of the system is proved by the Lyapunov theory.

### 4.1. Nonsingular Fast Terminal Sliding Mode Controller Design

Generally, the theoretical design of sliding mode controller is based on Lyapunov stability theory. Programmatically, the design process is divided into three parts, i.e., (i) An appropriate sliding surface design; (ii) The equivalent control law design; (iii) The switching control law design. 

Step 1: Similar to [26], the NFTSMC surface s is selected as follows.

(16) $$s=k_{0}e_{1}+k_{1}|e_{1}{|}^{\alpha }\mathrm{sign}(e_{1})+k_{2}|e_{2}{|}^{\beta }\mathrm{sign}(e_{2})$$

where, $k_{0}$, $k_{1}$, $k_{2}$, α, and β are all positive constants, satisfying 1<β<2 and α>β. 

It can be seen from the surface Equation (16), for any given initial condition ${\left[e_{1}(0),e_{2}(0)\right]}^{T}\neq {\left[0,0\right]}^{T}$, the state can converge very quickly to the equilibrium state ${\left[0,0\right]}^{T}$ in finite time. Moreover, among the parts of $k_{0}e_{1}$, $k_{1}|e_{1}{|}^{\alpha }\mathrm{sign}(e_{1})$ , and $k_{2}|e_{2}{|}^{\beta }\mathrm{sign}(e_{2})$, it is not difficult to get that, when the state is far from the equilibrium state, the subitem $k_{1}|e_{1}{|}^{\alpha }\mathrm{sign}(e_{1})$ dominates and ensures the faster convergence rate. Meanwhile, when the state is close to the equilibrium state, the subitem $k_{2}|e_{2}{|}^{\beta }\mathrm{sign}(e_{2})$ also can guarantee the system convergence in a finite time.

Step 2: From Equations (13) and (16), the first derivative of sliding mode surface s is calculated as follows.

(17) $$\dot{s}=k_{0}e_{2}+k_{1}\alpha |e_{1}{|}^{\alpha -1}e_{2}+k_{2}\beta |e_{2}{|}^{\beta -1}(-{\ddot{\theta }}_{d}+A_{0}\dot{\theta }+B_{0}u+\delta )$$

It is clear that the condition $\dot{s}\;=0$ is necessary for the state trajectory to stay on sliding surface s=0. Thus, from Equation (17), do not consider the lumped disturbance, the equivalent control law can be obtained as

(18) $$u_{\mathrm{eq}}=\frac{1}{B_{0}}[{\ddot{\theta }}_{d}-A_{0}\dot{\theta }-\frac{1}{k_{2}\beta }|e_{2}{|}^{2-\beta }(k_{0}+k_{1}\alpha |e_{1}{|}^{\alpha -1})\mathrm{sign}(e_{2})]$$

Step 3: In fact, the equivalent control law $u_{\mathrm{eq}}$ only can make the system state stay on the sliding surface if the dynamic model is known exactly. However, due to the presence of the unmodelled parts and uncertain external disturbances in practical applications, the accuracy of the control system is impossible to guarantee only by the equivalent control.

So, after choosing the equivalent control law, the next step is to design the switching control law which handles the lumped disturbances, here, an exponential reaching rate is selected as follows.

(19) $$\dot{s}=-\mathrm{ks}-(D+\eta )\mathrm{sign}(s)$$

where the switching gain k is a positive constant, and η is a small positive constant.

Then, from Equations (15) and (17), the switching control law is derived as

(20) $$u_{\mathrm{sw}}=\frac{1}{B_{0}}[-\mathrm{ks}-(d_{0}+d_{1}||e_{1}||+d_{2}||e_{2}|{|}^{2}+\eta )\mathrm{sign}(s)]$$

Hence, the total control law $u_{\mathrm{NFTSMC}}$ of the NFTSMC is the sum of the equivalent control law $u_{\mathrm{eq}}$ and the switching control law $u_{\mathrm{sw}}$ by the Equations (18) and (20).

(21) $$\begin{array}{ll} u_{\mathrm{NFTSMC}}= & \frac{1}{B_{0}}[{\ddot{\theta }}_{d}-A_{0}\dot{\theta }-\frac{1}{k_{2}\beta }|e_{2}{|}^{2-\beta }(k_{0}+k_{1}\alpha |e_{1}{|}^{\alpha -1})\mathrm{sign}(e_{2})\\ & -(d_{0}+d_{1}||e_{1}||+d_{2}||e_{2}|{|}^{{}^{2}}+\eta )\mathrm{sign}(s)-\mathrm{ks}] \end{array}$$

### 4.2. Adaptive Controller Design

Generally, in the procedure of the designation for NFTSMC, in order to guarantee the robustness of the control system, a lager switching gain should be selected conservatively. Although a larger gain can ensure the system convergence, it often inevitably causes chattering. Thus, based on adaptive control theory, we can choose ${\hat{d}}_{0}$, ${\hat{d}}_{1}$, and ${\hat{d}}_{2}$ to estimate the uncertain parameters $d_{0}$, $d_{1}$, and $d_{2}$, respectively. Then, from Equation (20) the adaptive switching control law $u_{\mathrm{asw}}$ is derived as follows.

(22) $$u_{\mathrm{asw}}=\frac{1}{B_{0}}[-\mathrm{ks}-({\hat{d}}_{0}+{\hat{d}}_{1}||e_{1}||+{\hat{d}}_{2}||e_{2}|{|}^{2}+\eta )\mathrm{sign}(s)]$$

Meanwhile, from Equation (21), the total control law $u_{\mathrm{ANFTSMC}}$ of the ANFTSMC is concluded as follows.

(23) $$\begin{array}{ll} u_{\mathrm{ANFTSMC}}= & \frac{1}{B_{0}}[{\ddot{\theta }}_{d}-A_{0}\dot{\theta }-\frac{1}{k_{2}\beta }|e_{2}{|}^{2-\beta }(k_{0}+k_{1}\alpha |e_{1}{|}^{\alpha -1})\mathrm{sign}(e_{2})\\ & -({\hat{d}}_{0}+{\hat{d}}_{1}||e_{1}||+{\hat{d}}_{2}||e_{2}|{|}^{2}+\eta )\mathrm{sign}(s)-\mathrm{ks}] \end{array}$$

Let define the adaptation error as ${\tilde{d}}_{0}={\hat{d}}_{0}-d_{0}$, ${\tilde{d}}_{1}={\hat{d}}_{1}-d_{1}$, ${\tilde{d}}_{2}={\hat{d}}_{2}-d_{2}$, the update laws of the estimation parameters ${\hat{d}}_{0}$, ${\hat{d}}_{1}$, and ${\hat{d}}_{2}$ can be designed as follows.

(24) $${\dot{\hat{d}}}_{0}=\mu_{0}|s|\cdot |e_{2}{|}^{\beta -1}$$

(25) $${\dot{\hat{d}}}_{1}=\mu_{0}|s|\cdot |e_{2}{|}^{\beta -1}||e_{1}||$$

(26) $${\dot{\hat{d}}}_{0}=\mu_{0}|s|\cdot |e_{2}{|}^{\beta -1}||e_{2}|{|}^{2}$$

where $\mu_{0}$, $\mu_{1}$, and $\mu_{2}$ are all positive constants.

### 4.3. A Novel Reaching Law Design

Generally, in the Equation (23), since a larger gain k is required to obtain a faster reaching performance, when a larger gain is inappropriately selected, it is usually lead to excessive speed when reaching the sliding surface, which probably causes serious chattering. 

For this reason, we redesign the reaching law Equation (19), in which the constant gain k is replaced by a modified normal distribution function $\bar{k}\;>0$ as follows.

(27) $$\bar{k}=\left\{ \begin{array}{ll} \lambda k_{a} & \mathrm{if}\;|e_{1}|\;\geq \sigma_{1}\;\mathrm{or}\;|e_{2}|\;\geq \sigma_{2}\\ k_{a}[1-\exp(-e_{1}^{2}/\sigma )]\;\; & \mathrm{if}\;|e_{1}|\;<\sigma_{1}\;\mathrm{and}\;|e_{2}|\;<\sigma_{2} \end{array}\right.$$

where $k_{a}$, λ, σ, $\sigma_{1}$, and $\sigma_{2}$ are all positive constants, satisfying λ>1.

Obviously, the switching gain $\bar{k}$ varying respect to the angular position error $e_{1}$, comparative with the Equation (19), the novel reaching law can accelerate convergence if the state is far from the sliding surface, but also further weaken the chattering when the state maintain on the sliding surface.

Hence, from Equations (23) and (27), the total control law $u_{\mathrm{ANFTSMC}}$ is rewritten as

(28) $$\begin{array}{ll} u_{\mathrm{ANFTSMC}}= & \frac{1}{B_{0}}[{\ddot{\theta }}_{d}-A_{0}\dot{\theta }-\frac{1}{k_{2}\beta }|e_{2}{|}^{2-\beta }(k_{0}+k_{1}\alpha |e_{1}{|}^{\alpha -1})\mathrm{sign}(e_{2})\\ & -({\hat{d}}_{0}+{\hat{d}}_{1}||e_{1}||+{\hat{d}}_{2}||e_{2}|{|}^{2}+\eta )\mathrm{sign}(s)-\bar{k}s] \end{array}$$

### 4.4. Stability Analysis

**Figure 3:**
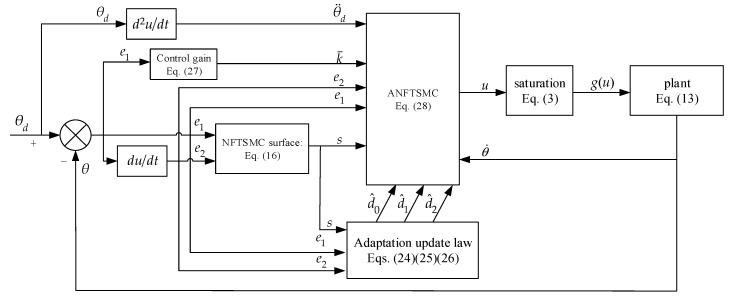
Block diagram of the proposed adaptive nonsingular fast terminal sliding mode control.

In this section, the Lyapunov stability theory is employed to analysis the stability of the control system. Such as in the Section 4.1 and Section 4.2, the block diagram of the ANFTSMC structure is presented in Figure 3.

We have the following theorem of the proposed ANFTSMC method:

**Theorem 1.**  *Consider the system Equation (1) with unknown upper bound of the lumped uncertain disturbances and non-smooth saturation input approximated by a smooth function, if the NFTSMC surface is selected as Equation (16), the adaptive controller is designed as Equation (23) and the adaptation laws are chosen as Equations (24)–(26), and the constant gain in the switching control law replaced by the Equation (27), then the system states can converge to the sliding surface in a finite time and maintain the system trajectory on it for the subsequent time.*

In general, the selection of the Lyapunov function and design of the adaptive law complement each other. As a matter of experience, especially in sliding mode control, Lyapunov function candidate can be divided as two parts, the basic part can be constructed by sliding surface, the rest part is the matching terms related to the adaptive law, which is selected by trial and error based on reverse deduction method.

For the sake of fluency, we use the positive sequence to state this part, i.e., firstly the adaptive law and the Lyapunov function candidate are selected and then the stability is proved.

**Proof.**  Consider the following Lyapunov function candidate:

(29) $$V=\frac{1}{2}s^{2}+k_{2}\beta \sum_{i=0}^{2}\frac{1}{2\mu_{i}}{\left({\hat{d}}_{i}-d_{i}\right)}^{2}$$

by differentiating V with respect to time yields

(30) $$\dot{V}=s\dot{s}+k_{2}\beta \sum_{i=0}^{2}\frac{1}{\mu_{i}}({\hat{d}}_{i}-d_{i}){\dot{\hat{d}}}_{i}$$

Considering Equations (17) and (28), then Equation (30) can be rewritten as follows

(31) $$\dot{V}=k_{2}\beta |e_{2}{|}^{\beta -1}[\delta s-\bar{k}s^{2}-({\hat{d}}_{0}+{\hat{d}}_{1}||e_{1}||+{\hat{d}}_{2}||e_{2}|{|}^{2}+\eta )|s|]+k_{2}\beta \sum_{i=0}^{2}\frac{1}{\mu_{i}}({\hat{d}}_{i}-d_{i}){\dot{\hat{d}}}_{i}$$

By substituting the adaptation update laws Equations (24)–(26), the time derivative of V leads to

(32) $$\begin{array}{ll} \dot{V}= & k_{2}\beta |e_{2}{|}^{\beta -1}[\delta s-\bar{k}s^{2}-({\hat{d}}_{0}+{\hat{d}}_{1}||e_{1}||+{\hat{d}}_{2}||e_{2}|{|}^{2}+\eta )|s|]\\ & +k_{2}\beta |s|\cdot |e_{2}{|}^{\beta -1}[({\hat{d}}_{0}-d_{0})+({\hat{d}}_{1}-d_{1})||e_{1}||+({\hat{d}}_{2}-d_{2})||e_{2}|{|}^{2}] \end{array}$$

Simplifying Equation (32) yields

(33) $$\dot{V}=k_{2}\beta |e_{2}{|}^{\beta -1}[\delta s-\bar{k}s^{2}-(d_{0}+d_{1}||e_{1}||+d_{2}||e_{2}|{|}^{2}+\eta )|s|]$$

From Equations (14) and (15), then Equation (33) can be concluded as

(34) $$\begin{array}{ll} \dot{V} & \leq k_{2}\beta |e_{2}{|}^{\beta -1}[|D|\cdot |s|-\bar{k}s^{2}-(d_{0}+d_{1}||e_{1}||+d_{2}||e_{2}|{|}^{2}+\eta )|s|]\\ & \leq k_{2}\beta |e_{2}{|}^{\beta -1}(-\bar{k}s^{2}-\eta |s|)\\ & \leq 0 \end{array}$$

This completes the proof.□

According to Lyapunov stability theorem, the system states converge to the surface s=0 asymptotically, i.e., even in the presence of system uncertainties and external disturbance, the system can converge and maintain on the surface from any initial condition states, thus ensuring the robustness of the system. Next, the convergence time of ANFTSMC will be discussed compared with [26] as follows.

Reconsidering Lyapunov function candidate Equation (29), the inequality (34) can be rewritten as

(35) $$\begin{array}{ll} \frac{\mathrm{dV}}{\mathrm{dt}} & \leq k_{2}\beta |e_{2}{|}^{\beta -1}(-\bar{k}s^{2}-\eta |s|)\\ & =-2\bar{k}k_{2}\beta |e_{2}{|}^{\beta -1}\left[V-k_{2}\beta \sum_{i=0}^{2}\frac{1}{2\mu_{i}}{\left({\hat{d}}_{i}-d_{i}\right)}^{2}\right]-\sqrt{2}\eta k_{2}\beta |e_{2}{|}^{\beta -1}{\left[V-k_{2}\beta \sum_{i=0}^{2}\frac{1}{2\mu_{i}}{\left({\hat{d}}_{i}-d_{i}\right)}^{2}\right]}^{1/2} \end{array}$$

By defining $\rho_{1}=2\bar{k}k_{2}\beta |e_{2}{|}^{\beta -1}$, $\rho_{2}=\sqrt{2}\eta k_{2}\beta |e_{2}{|}^{\beta -1}$, and $\nabla =k_{2}\beta \sum_{i=0}^{2}\frac{1}{2\mu_{i}}{\left({\hat{d}}_{i}-d_{i}\right)}^{2}$, then inequality (35) leads to

(36) $$\begin{array}{ll} \mathrm{dt} & \leq \frac{-\mathrm{dV}}{\rho_{1}(V-\nabla )+\rho_{2}{\left(V-\nabla \right)}^{1/2}}\\ & =\frac{-{\left(V-\nabla \right)}^{1/2}\mathrm{dV}}{\rho_{1}{\left(V-\nabla \right)}^{1/2}+\rho_{2}}=-2\frac{d{\left(V-\nabla \right)}^{1/2}}{\rho_{1}{\left(V-\nabla \right)}^{1/2}+\rho_{2}} \end{array}$$

Suppose that the reaching time from the initial state $V_{t_{0}}\neq 0$ to the equilibrium state $V_{t_{f}}=0$ is $t_{f}$. Taking integral of both sides of the inequality (36) yields

(37) $$t_{f}\leq -\frac{2}{\rho_{1}}\ln\left[\frac{\rho_{1}{\left(V_{t_{f}}-\nabla_{t_{f}}\right)}^{1/2}+\rho_{2}}{\rho_{2}}\right]+\frac{2}{\rho_{1}}\ln\left[\frac{\rho_{1}{\left(V_{t_{0}}-\nabla_{t_{0}}\right)}^{1/2}+\rho_{2}}{\rho_{2}}\right]$$

Furthermore in Lyapunov function candidate Equation (29), it is not difficult to get the following implicit relationships

(38) $$V_{t}\geq V_{t}-\nabla_{t}=\frac{1}{2}s_{t}^{2}\geq 0,\;\forall t\in [t_{0},t_{f}]$$

(39) $$V_{t_{f}}-\nabla_{t_{f}}=\frac{1}{2}s_{t_{f}}^{2}=0$$

Then, combining the inequalities (38) and (39), the inequality (36) concludes as

(40) $$\begin{array}{ll} t_{f} & \leq \frac{2}{\rho_{1}}\ln\left[\frac{\rho_{1}{\left(V_{t_{0}}-\nabla_{t_{0}}\right)}^{1/2}+\rho_{2}}{\rho_{2}}\right]\\ & =\frac{2}{\rho_{1}}\ln\left[\frac{\sqrt{2}\rho_{1}s_{t_{0}}}{2\rho_{2}}+1\right] \end{array}$$

Comparison with [26], the convergence time of the ANFTSMC is the same as the ones by the NFTSMC, i.e., it is concluded that the introduction of the adaptive controller Equation (28) does not change the finite convergence time by NFTSMC.

## 5. Simulation and Experimental Results

In this section, in order to verify the effectiveness of the proposed ANFTSMC strategy, extensive simulations and practical experiments are carried out. As mentioned in the beginning of Section 3, the motion of each axis of the UISP can be considered independently. Each axis can be designed separately according its own motor parameters. It is worth mentioning that the cantilever structure of the A-gimbal makes it more vulnerable to the disturbance of imbalance and fiction torques, at the same time, considering the limited space, without loss of generality, only the A-gimbal is selected to validate the proposed ANFTSMC method. In the practical applications, the precision of the low-speed tracking is higher than that of the large-angle swing canning, in order to enhance the comparability of our research, all the control algorithms are designed based on low-speed tracking under the premise of giving priority to ensure its good control performance, and then the same control parameters are applied to the large-angle swing scanning. In addition, due to the attitude compensation cannot be realized in the simulation, in order to ensure the fairness of the comparison between simulation and experiment, the attitude compensation was not added to the experiment presented in this paper.

### 5.1. Simulation Results and Discussions

The simulation is carried out by MATALB/Simulink based on the block diagram of the proposed ANFTSMC shown in the Figure 3 and the parameters of the A-gimbal DC motor listed in Table 1.

In the simulation, the initial conditions and parameters of the system are set as ${\left[\theta (0),\dot{\theta }(0)\right]}^{T}={\left[0,0\right]}^{T}$, $J_{0}=0.255\;\mathrm{kg}\cdot m^{2}$, $A_{0}=-0.5$, $B_{0}=25$, the maximum output of the actuator is $\varphi_{\max}=28$, and the uncertain disturbances including both low and high frequency signals are selected as δ=sin(0.01πt)+cos(2t)+sin(50πt); in addition, external disturbances contains step signal and random signal are selected as $\delta_{\mathrm{add}}=1+\mathrm{rand}(1)$ are added to the system at time t≥0.6 s, the sampling time is t=1 ms. Meanwhile, due to the variation range of the moment of inertia $J_{0}$ is $0.227\sim 0.281\;\mathrm{kg}\cdot m^{2}$, so in order to validate robustness of the proposed method, the condition of deviation ±15% about $J_{0}$ base on $J_{0}=0.255\;\mathrm{kg}\cdot m^{2}$ is also considered. The following four different control methods are tested and compared in the simulations.

**Method 1.**  *The traditional PID controller. Since the system has not established an exact dynamic model, the controller gains are selected as* $K_{p}=1000$ *,* $K_{i}=5$ *,* $K_{d}=5$ *by trial and errors according to the order of Kp, Ki and Kd.*

**Method 2.**  *The NFTSMC with a variable gain. The controller is expressed as Equation (21), and taking variable gain*$\bar{k}$*Equation (27) replace constant gain*k*in the Equation (21), set*α=2*,*β=1.99*,*$k_{0}=1$*,*$k_{1}=100$*,*$k_{2}=0.5$*,*$d_{0}=10$*,*$d_{1}=5$*,*$d_{2}=2$*,*η=1*,*$k_{a}=20$*,*λ=100*,*σ=0.1*,*$\sigma_{1}=0.01$*,*$\sigma_{2}=0.01$.

**Method 3.**  *The ANFTSMC with a small constant gain. The controller is expressed as Equation (23), set*α=2*,*β=1.99*,*$k_{0}=1$*,*$k_{1}=100$*,*$k_{2}=0.5$*,*$\mu_{0}=0.1$*,*$\mu_{1}=0.1$*,*$\mu_{2}=0.1$*,*η=1*,*k=200.

**Method 4.**  *The proposed ANFTSMC method. The controller is expressed as Equation (28), set*α=2*,*β=1.99*,*$k_{0}=1$*,*$k_{1}=100$*,*$k_{2}=0.5$*,*$\mu_{0}=0.1$*,*$\mu_{1}=0.1$*,*$\mu_{2}=0.1$*,*η=1*,*$k_{a}=20$*,*λ=100*,*σ=0.1*,*$\sigma_{1}=0.01$*,*$\sigma_{2}=0.01$.

The maximum absolute error (MAE) and the steady-state root mean square (RMS) are used to evaluate the performances of different control methods.

The definition of MAE is as follows:

(41) $$\mathrm{MAE}=\max|e_{i}|$$

The definition of RMS is as follows:

(42) $$\mathrm{RMS}={\left[\frac{1}{n-1}\sum_{i=1}^{n}{\left(x_{i}-\bar{x}\right)}^{2}\right]}^{1/2}$$

where $x_{i}$ is the sample data, $\bar{x}$ is the mean value of the sample data $x_{i}$.

#### 5.1.1. Case 1—Sinusoidal Signal Tracking

**Figure 4:**
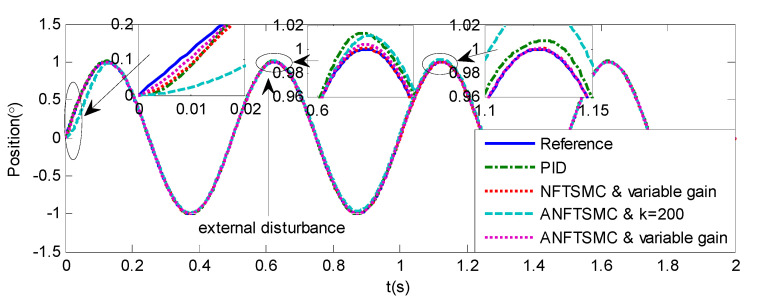
Simulation of A-gimbal system—Angular position tracking responses to sinusoidal signal.

**Figure 5:**
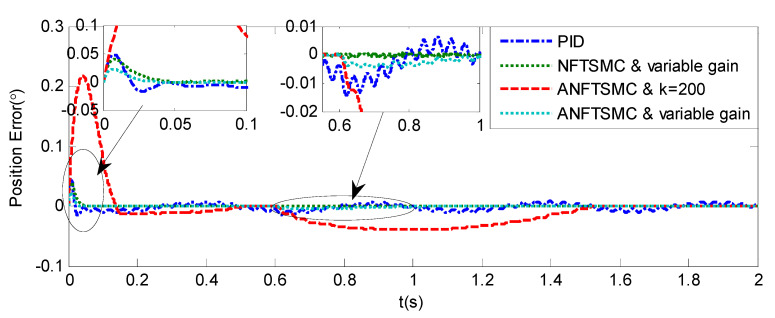
Simulation of A-gimbal system—Angular position tracking error responses to sinusoidal signal.

**Figure 6:**
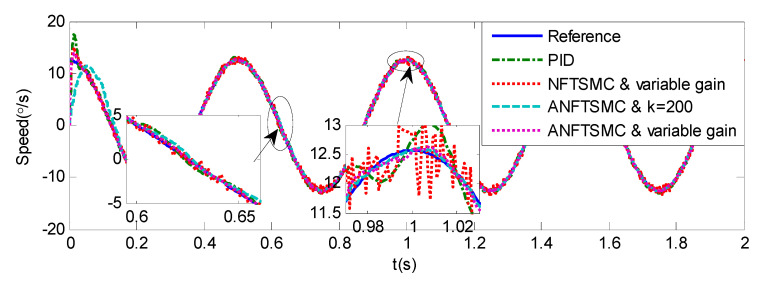
Simulation of A-gimbal system—Angular velocity tracking responses to sinusoidal signal.

**Figure 7:**
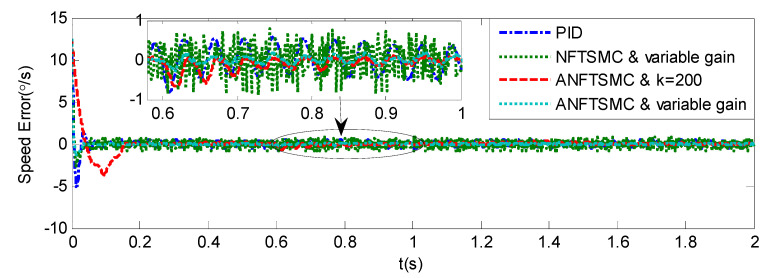
Simulation of A-gimbal system—Angular velocity tracking error responses to sinusoidal signal.

**Figure 8:**
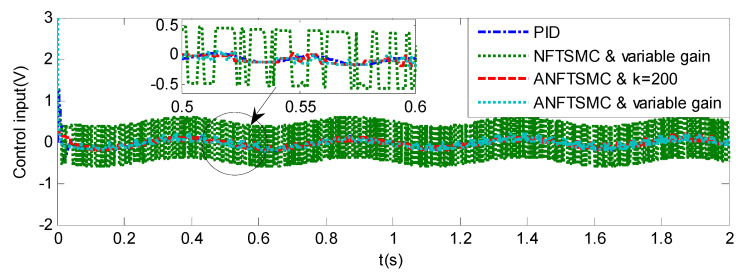
Simulation of A-gimbal system—Control Input responses to sinusoidal signal.

**Figure 9:**
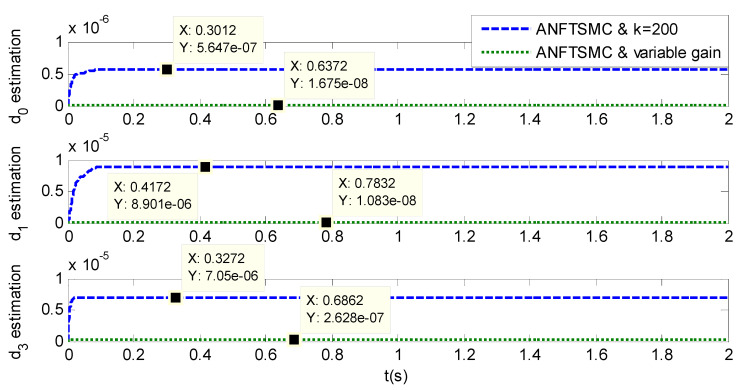
Simulation of A-gimbal system—Estimation of the parameters of disturbances by two different ANFTSMC.

**Figure 10:**
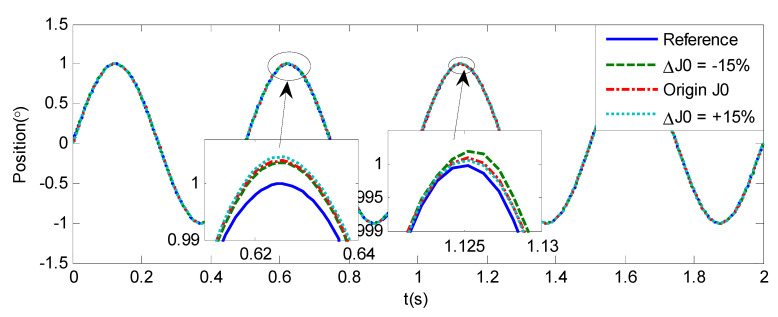
Simulation of A-gimbal system—Angular position tracking responses to sinusoidal signal by proposed method under the condition of deviation about J0.

**Figure 11:**
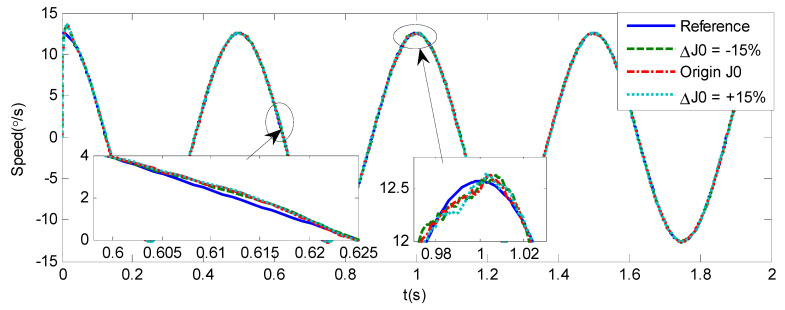
Simulation of A-gimbal system—Angular velocity tracking responses to sinusoidal signal by proposed method under the condition of deviation about J0.

Sinusoidal Signal Tracking can reflect the low-speed video tracking performance, the reference signal is selected as $\theta_{d}=\sin(4\pi t)$. The comparative simulation results are shown in Figure 4, Figure 5, Figure 6, Figure 7, Figure 8, Figure 9, Figure 10 and Figure 11. The performance indexes of simulation results as shown in Table 2.

As shown in Figure 4, Figure 5, Figure 6 and Figure 7 and Table 2, it can be seen that all the four methods can track the desired position and speed signals with the lumped uncertain disturbances. From the MAE and RMS, we know that the position and speed tracking errors of the proposed ANFTSMC method are much smaller compared with those of PID and NFTSMC. Moreover, by using variable gain, the convergence time of the proposed ANFTSMC method can be reduced to 0.07 s from 0.50 s, and the external disturbances appear suddenly, the propose method can convergence again faster than the ANFTSMC with the constant gain. Specifically, as shown in Figure 6, Figure 7 and Figure 8, the chattering of the proposed ANFTSMC method is much smaller compared with that of the NTFSMC. As shown in Figure 9, the estimation parameters in the proposed method are smaller than those by ANFTSMC with the constant gain, due to the adaptive laws are related to the state errors, it reveals that the convergence speed is faster by the proposed method. As shown in Figure 10 and Figure 11, the robustness of the proposed method has been validated under the condition of deviation about J0. In addition, it is worth noting that only PID controller has phase lag, if we want to reduce the phase lag, we need to increase the gain $k_{d}$. However, an inappropriate larger $k_{d}$ will easily excite the sensor noise, which is not allowed in the actual system.

Combining with above analysis, the proposed method can achieve much better low-speed tracking performance and transient response. 

#### 5.1.2. Case 2—Step Signal Tracking

**Figure 12:**
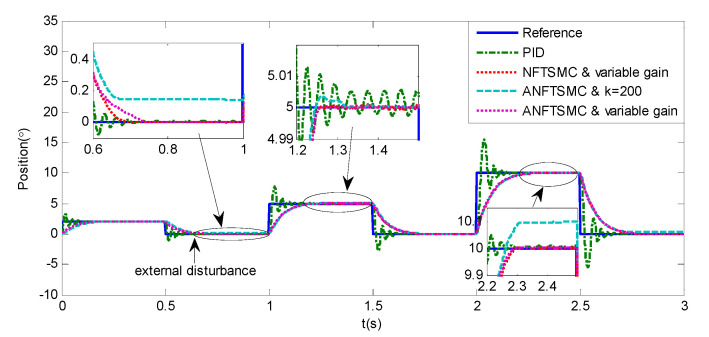
Simulation of A-gimbal system—Angular position tracking responses to step signal.

**Figure 13:**
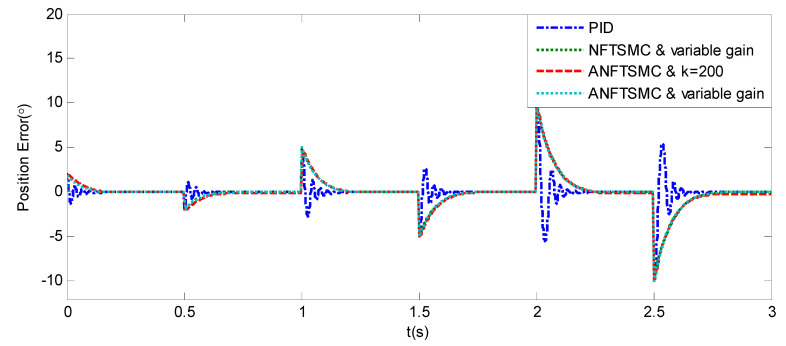
Simulation of A-gimbal system—Angular position tracking error responses to step signal.

**Figure 14:**
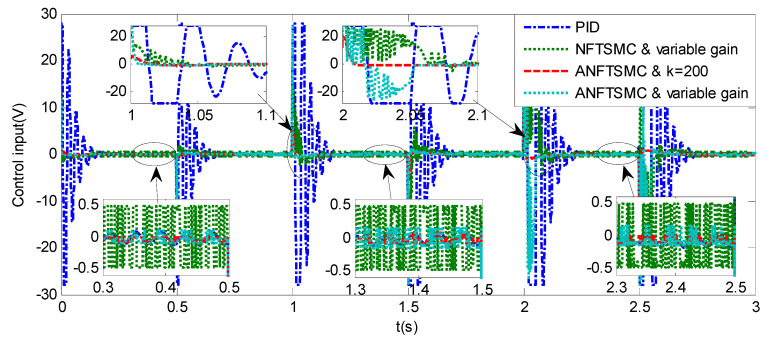
Simulation of A-gimbal system—Control Input responses to step signal.

Step Signal Tracking can reflect the large-angle stepping swing scanning, in this case, the system state error is expected to converge to zero in a desired time from far away. The reference signal is selected as Equation (43), and the comparative simulation results of the four methods are shown in Figure 12, Figure 13 and Figure 14. The performance indexes of simulation results as shown in Table 3.

(43) $$\theta_{d}=\{\begin{array}{ccc} 2{}^{\circ} & if & 0\leq t\leq 0.5\\ 5{}^{\circ} & if & 1\leq t\leq 1.5\\ 10{}^{\circ} & if & 2\leq t\leq 2.5\\ 0 & & else \end{array}$$

As shown in the Figure 12, Figure 13 and Figure 14 and the Table 3, except for the PID controller, in spite of different amplitudes of desired step signals, the other three methods based NFTSMC have no overshoot, furthermore, the proposed ANFTSMC method has the smallest RMS value and chattering. When the external disturbances appear suddenly, the proposed method with the variable gain can convergence again faster than that in ANFTSMC with a constant gain method.

**Figure 15:**
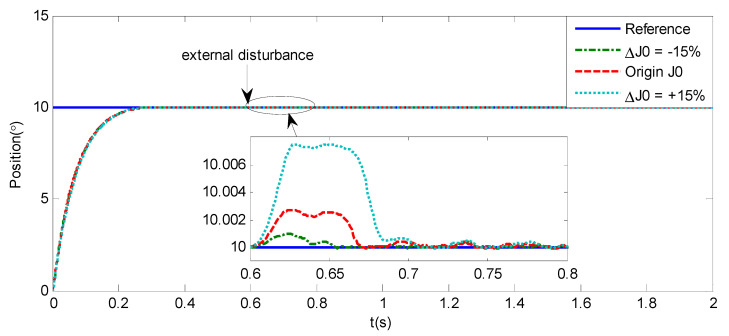
Simulation of A-gimbal system—Angular position tracking responses to step signal with amplitude of 10° under the condition of deviation about J0.

In order to verify the robustness of the proposed method, under the condition about J0, an additional step simulation is tested with the amplitude of 10°. As shown in Figure 15, if the external disturbances appear suddenly, the proposed method has good anti-disturbance performance and robustness.

Combining with above analysis, the proposed method can achieve much better large-angle step tracking performance and transient response.

### 5.2. Experimental Results and Discussions

**Figure 16:**
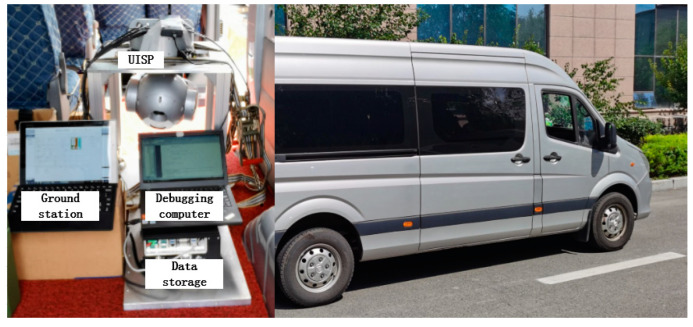
The picture of experimental system for the moving vehicle.

**Figure 17:**
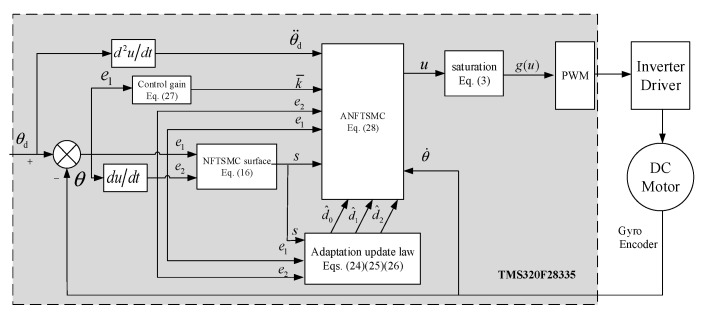
The equivalent circuit diagram of the A-gimbal in the universal inertially stabilized platform.

As shown in Figure 16, in order to validate the effectiveness of the proposed method for the three-axis UISP system, the moving vehicle experiments are carried out on the express way in Jilin Province of China. The UISP is mounted on the inner floor of the vehicle. In our experiments, it consists of the UISP, 28 V DC power, ground station, personal debugging computer and data storage. The equivalent circuit diagram of the UISP system is shown in Figure 17, all the control algorithms are written by C language downloaded to the DSP TMS320F28335.

In the experiments, the parameters of the system are set as $J_{0}=0.255\;\mathrm{kg}\cdot m^{2}$, $A_{0}=-0.5$, $B_{0}=25$, the maximum output of the actuator is $\varphi_{\max}=28$, and the sampling time is 1 ms. The encoder resolution is about 6.866 × 10^−4^ (deg.), and the gyro resolution is about 2.384 × 10^−5^ (deg./s). 

Similar to the Section 5.1, in order to further validate the control performance of the proposed ANFTSMC method, comparative experiments are carried out, as compared with PID control and NFTSMC. Since we have referred to the motor parameters in the simulation, so the parameters in the experiments are similar as those of the simulation, they only need to be fine-tuned according to the actual situation.

**Method 1.**  *PID Controller. In order to reduce the sensor noise, Kd is reduced accordingly. The parameters are selected as*$K_{p}=1000$*,*$K_{i}=5$*,*$K_{d}=0.5$.

**Method 2.**  *NFTSMC method with a constant gain. The parameters are select as*α=2*,*β=1.99*,*$k_{0}=1$*,*$k_{1}=100$*,*$k_{2}=0.5$*,*$d_{0}=10$*,*$d_{1}=5$*,*$d_{2}=2$*,*k=2000.

**Method 3.**  *Proposed ANFTSMC method. The parameters are select as*α=2*,*β=1.99*,*$k_{0}=1$*,*$k_{1}=100$*,*$k_{2}=0.5$*,*$\mu_{0}=0.1$*,*$\mu_{1}=0.1$*,*$\mu_{2}=0.1$*,*η=1*,*$k_{a}=20$*,*λ=100*,*σ=0.1*,*$\sigma_{1}=0.1$*,*$\sigma_{2}=0.5$.

#### 5.2.1. Case 1—Sinusoidal Signal Tracking

**Figure 18:**
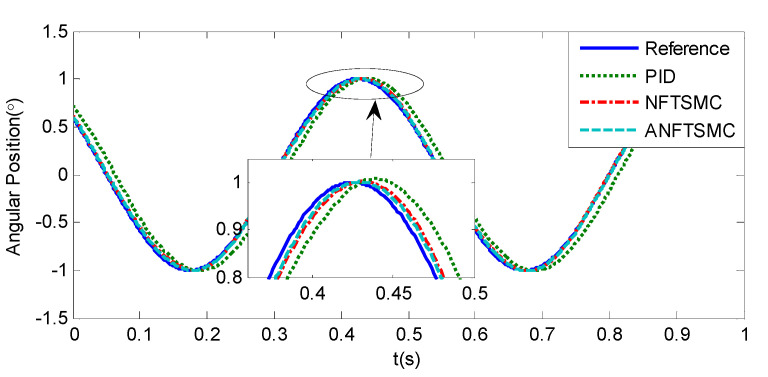
Practical experiments of A-gimbal system—Angular position tracking responses to sinusoidal signal.

**Figure 19:**
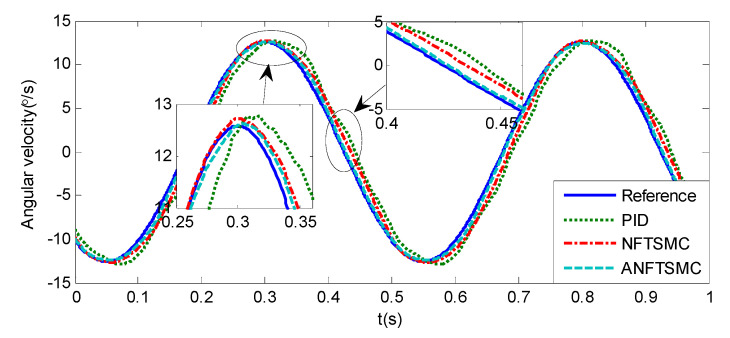
Practical experiments of A-gimbal system—Angular velocity tracking responses to sinusoidal signal.

In this Case, the desired signal is selected as $\theta_{d}=\sin(4\pi t)$, and the comparative experiment results are as show in the Figure 18 and Figure 19. The performance indexes of the control responses are shown in the Table 4.

As the results in the Table 4, by ignoring the inherent delay effect of the system, the proposed method has the smallest MAE and RMS value of both angular position and speed tracking. A particular attention is paid to the Figure 15, we can get that the phenomenon of stick-slip caused by friction only appears in the PID controller. So, the proposed method has better performances in the low-speed tracking application.

#### 5.2.2. Case 2—Step Signal Tracking

**Figure 20:**
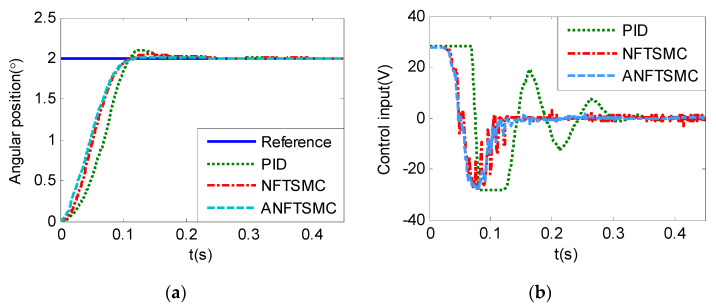
Practical experiments of A-gimbal system—Tracking responses to 2° step signal: (**a**) Angular position; (**b**) Control input.

**Figure 21:**
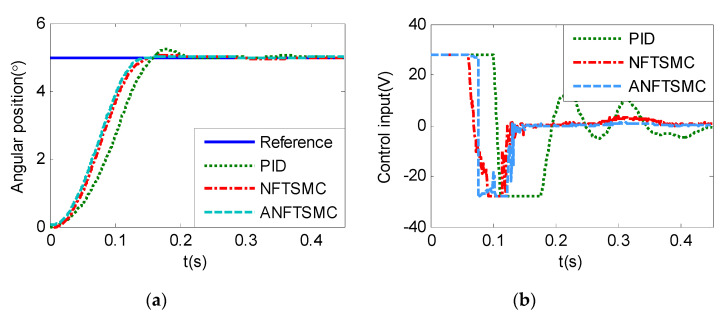
Practical experiments of A-gimbal system—Tracking responses to 5° step signal: (**a**) Angular position; (**b**) Control input.

**Figure 22:**
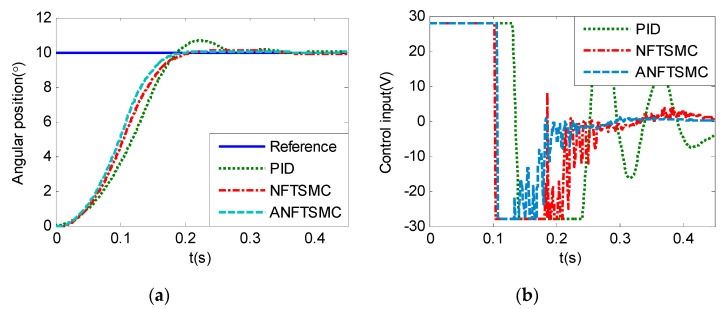
Practical experiments of A-gimbal system—Tracking responses to 10° step signal: (**a**) Angular position; (**b**) Control input.

In this case, the desired signal is set as $\theta_{d}=2{}^{\circ}(t\geq 0)$, $\theta_{d}=5{}^{\circ}(t\geq 0)$, and $\theta_{d}=10{}^{\circ}(t\geq 0)$, respectively, the comparative experiment results are as show in the Figure 20, Figure 21 and Figure 22. The performance indexes of the control responses are shown in the Table 5.

As the results in the Table 5, by ignoring the inherent delay effect of the system, if the initial state is far from the sliding surface, the rising time of NFTSMC and the proposed ANFTSMC methods is shorter than that of the PID controller, and the overshoot of the proposed ANFTSMC method is smallest. So, the proposed method is superiority in the large-angle step tracking application.

### 5.3. Discussions

Although the comparative simulations and experiments are carried out, some differences are obvious. What we have to explain is that the lower limit value of the DC motor parameters listed in the Table 1 was selected in the simulation; however, in the practical experiment, the actual torque parameters of the motor were better than those set in the simulation. So, in the step signal tracking, the methods have better transient performances. What is more, in the practical experiment by the PID method, in order to reduce the adverse effect of the sensor noises, under the sacrifice of the phase lag, the gain Kd was reduced from 5 to 0.5, and the performances improved more than those in the simulation.

## 6. Conclusions

In this paper, aiming at the practical engineering applications, including so called low-speed video tracking and large-angle swing scanning imaging, we proposed an adaptive nonsingular fast terminal sliding mode control strategy for a cantilever three-axis inertially stabilized platform subjected to the uncertain disturbances and input saturation constraints, so as to obtain better control performances. First of all, a second-order dynamic model with friction and torques, unmodelled dynamics, external disturbances, and input saturation constraints was established, where the non-smooth input saturation dynamic was approximated by a smooth hyperbolic tangent function, which makes it more convenient in engineering implementation. Based on the dynamic model, the proposed method inherits the advantage of the NFTSMC, which can drive the system state error to zero in finite time without singularity. In addition, a novel reaching law based on a normal distribution function was designed to adjust the control gain according to the position and speed errors, which not only accelerate convergence when the system state is far away from the equilibrium but also weaken the chattering when the state maintains on the sliding surface. This can obtain a better performance of low-speed video tracking and large-angle swing scanning applications at the same. It is worth mentioning that the proposed method can online estimate the parameters of the lumped uncertain disturbances, where the prior knowledge of the upper bounds is not required during the procedure of the controller design, which makes it more suitable to be applied in practical engineering. Finally, extensive comparative simulations and practical experiments have validated the effectiveness and superiority of the proposed ANFTSMC method, the results show the proposed method has much better tracking performance and transient response.

## Figures and Tables

**Table 1:** Parameters of the A-gimbal DC torque motor.

Description	Value
Rated voltage	28 V
Peak torque	≥8.2 N·m
Peak current	5 A
Continuous plugging torque	≥4.0 N·m
Continuous plugging current	2.4 A
Speed (Max. no-load)	150 r/min
Torque ripple coefficient	3.27%
Armature resistance	5.45 Ω
Armature inductance	3.57 mH
Moment of inertia	0.227–0.281 kg·m^2^

**Table 2:** The simulation results of sinusoidal signal tracking for the A-gimbal system.

Method	Δ J0	Position Error (deg.)		Speed Error (deg./s)		Convergence	Phase
		MAE	RMS	MAE	RMS	Time (s)	Lag (rad)
PID	0	1.42 × 10^−2^	5.2 × 10^−3^	0.8275	0.3332	0.14	0.0754
NFTSMC and Variable gain	0	9.97 × 10^−4^	3.79 × 10^−4^	0.9256	0.3670	0.05	No
ANFTSMC and k = 200	0	3.97 × 10^−2^	2.20 × 10^−2^	0.6703	0.1266	0.50	No
Proposed	0	1.06 × 10^−3^	5.41 × 10^−4^	0.2889	0.0607	0.07	No
	−15%	1.19 × 10^−3^	5.58 × 10^−4^	0.3171	0.0622	0.07	No
	+15%	1.11 × 10^−3^	5.42 × 10^−4^	0.3045	0.0618	0.07	No

**Table 3:** The simulation results of step signal tracking for the A-gimbal system.

Method	Desired Angle (deg.)	Rising Time(s)	Setting Time(s)	Overshoot (%)	Steady-State RMS (deg.)
PID	2	0.011	0.110	63.2	3.368 × 10^−3^
NFTSMC and Variable gain	2	0.110	0.132	0	2.779 × 10^−3^
ANFTSMC and k = 200	2	0.112	0.138	0	6.415 × 10^−3^
Proposed Method	2	0.110	0.132	0	1.926 × 10^−3^
PID	5	0.012	0.113	54.2	3.718 × 10^−3^
NFTSMC and Variable gain	5	0.131	0.139	0.06	3.361 × 10^−4^
ANFTSMC and k = 200	5	0.133	0.142	0	7.817 × 10^−4^
Proposed Method	5	0.131	0.138	0	2.154 × 10^−4^
PID	10	0.017	0.124	54.4	3.599 × 10^−3^
NFTSMC and Variable gain	10	0.133	0.141	0.73	4.584 × 10^−4^
ANFTSMC and k = 200	10	0.151	0.177	0	8.127 × 10^−2^
Proposed Method	10	0.134	0.141	0	2.533 × 10^−4^

**Table 4:** The practical experiments results of sinusoidal signal tracking for the A-gimbal system.

Method	Position (deg.)		Speed (deg./s)		Phase Lag (rad.)
	MAE	RMS	MAE	RMS	
PID	0.1219	0.0857	3.258	1.8316	0.0943
NFTSMC	0.0506	0.0343	1.0867	0.6662	0.0377
Proposed Method	0.0348	0.0204	0.5013	0.2643	0.0251

**Table 5:** The practical experiments results of step signal tracking for the A-gimbal system.

Method	Desired Angle (deg.)	Rising Time (s)	Setting Time (s)	Overshoot (%)	Steady-State RMS (deg.)
PID	2	0.070	0.136	5.250	0.0354
NFTSMC	2	0.057	0.095	2.645	0.0227
ANFTSMC	2	0.062	0.094	1.012	0.0114
PID	5	0.096	0.178	4.628	0.0570
NFTSMC	5	0.085	0.131	1.328	0.0307
ANFTSMC	5	0.083	0.124	0.584	0.0159
PID	10	0.116	0.243	6.714	0.0740
NFTSMC	10	0.112	0.177	1.308	0.0426
ANFTSMC	10	0.105	0.165	0.556	0.0189
